# Humanizing birth in Tanzania: a qualitative study on the (mis) treatment of women during childbirth from the perspective of mothers and fathers

**DOI:** 10.1186/s12884-019-2385-5

**Published:** 2019-07-05

**Authors:** Lilian T. Mselle, Thecla W. Kohi, Justine Dol

**Affiliations:** 1 Department of Clinical Nursing, Muhimbili University of Health and Allied Sciences, Dar es Salaam, Tanzania; 2 School of Medicine, St. Joseph College of Health and Allied Sciences, Dar es Salaam, Tanzania; 3 Faculty of Health, Dalhousie University, Halifax, Canada

**Keywords:** Respectful maternity care, Birth care, Humanizing birth, Qualitative, Tanzania, Healthcare facilities, Mistreatment

## Abstract

**Background:**

While there has been a trend for greater number of women to deliver at health facilities across Tanzania, mothers and their family members continue to face mistreatment with respectful maternity care during childbirth being violated. The objective of this study was to describe the experience of mothers and fathers in relation to (mis) treatment during childbirth in Tanzania.

**Methods:**

Using a qualitative descriptive design, 12 semi-structured interviews and four focus group discussions were held with mothers and fathers who were attending a postnatal clinic in the Lake Zone region of Tanzania. Mothers’ age ranged from 20 to 45 years whereas fathers’ age ranged from 25 to 60 years. Data were analyzed using a priori coding based on Bohren’s et al. typology of the mistreatment of women during childbirth.

**Results:**

Mothers reported facing mistreatment and disrespectful maternity care through verbal abuse (harsh or rude language and judgmental or accusatory comments), failure to meet professional standards of care (refused pain relief, unconsented surgical operations, neglect, abandonment or long delays, and skilled attendant absent at time of delivery), poor rapport between women and providers (poor communication, lack of supportive care, denied husbands presence at birth, denied mobility, denied safe traditional practices, no respect for their preferred birth positions), and health system conditions and constraints (poor physical condition of facilities, supply constraints, bribery and extortion, unclear fee structures). Despite some poor care, some mothers also reported positive birthing experiences and respectful maternity care by having a skilled attendant assistance at delivery, having good communication from nurses, receiving supportive care from nurses and privacy during delivery.

**Conclusion:**

Despite the increasing number of deliveries occurring in the hospital, there continue to be challenges in providing respectful maternity care. Humanizing birth care in Tanzania continues to have a long way to go, however, there is evidence that changes are occurring as mothers notice and report positive changes in delivery care practices.

**Electronic supplementary material:**

The online version of this article (10.1186/s12884-019-2385-5) contains supplementary material, which is available to authorized users.

## Background

Respectful maternity care and humanizing the birth process is being increasingly emphasised in Tanzania by encouraging healthcare providers to care for women in a way that respects their rights as human beings. Respectful maternity care ensures provision of sufficient and adequate care throughout the birth process, involving women as active agents and capable of making decisions [1]. It emphasizes the fundamental right of mothers, newborns, and their families, while recognizing that all childbearing women need and deserve respectful care and protection of their rights [2]. Respectful maternity care promotes nine key attributes including; (i) respect for beliefs, traditions, and culture, (ii) empowerment of the women and her family to become active participants in health care, (iii) continuous support during childbirth, (iv) freedom of movement during labour, (v) choice of position during birth, (vi) good communication between client and provider, (vii) support of the mother-baby-pair, (viii) improvement of working conditions and respectful and collaborative relationships among all cadres of health workers and (ix) prevention of disrespect and abuse and institutional violence against women [2]. In order to humanize the birth process, women should be informed of what is happening, be able to participate in the decision making process, and fully understand their diagnosis and treatment options [1].

Disrespect and abuse during facility-based childbirth has been found to have negative effects on skilled birth care utilization. Women may choose not to seek care from a provider who does not treat them well, even if the provider is skilled at preventing and managing complications [2–4]. It is not uncommon to hear stories from mothers and their family members that rights to respectful maternity care have been violated, not only in Tanzania but around the world [5]. The continued ineffective and incomplete implementation of non-humanized birth care disfranchises women to deliver at health facilities. As a result, women in Tanzania struggle with the decision about where to give birth with poor care often pushing them away from delivering in the healthcare facility with a skilled birth attendant, despite increased risk of fatal newborn and maternal outcomes [4, 6].

The Tanzania Vision 2025 outline the goals of providing “access to quality reproductive health services for all individuals of appropriate ages” and “reduction in infant and maternal mortality rates by three-quarters of current levels” by 2025 [7]. Without improving the quality of care received at such facilities and understanding why women chose to deliver at facilities, attaining these goals may not be possible. One of the opportunities to reduce infant and maternal mortality is through increased deliveries at a healthcare facility [8]. While women are increasingly to deliver at health facilities across Tanzania and the rate has increased from 44% in 1999 to 63 in 2016, this has been found to vary depending on parity, age, geographic location, and the education level of prospective mothers [9]. Our previous work has shown that ﻿systematic barriers and institutional norms and practices limit midwives ability to provide respectful maternity care [10], however, women and men wish to deliver at health facilities if improvements can be made [6]. In order to explore the experiences of mothers and fathers on humanizing birth care in Tanzanian health facilities, the Bohren’s et al. typology of the mistreatment of mothers during childbirth in health facilities was used [11]. Using this typology to guide the analysis allows the Tanzanian experience to emerge among the broader picture of mothers’ childbirth experience across 34 countries.

## Objective

This descriptive qualitative study describes the experience of mothers and fathers on humanizing birth care in Tanzania using Bohren’s et al. [11] typology of the mistreatment as an a priori guiding framework.

## Methods

### Study design & setting

This study was part of a larger project exploring community and skilled health personnel perceptions and practices on humanizing birth care in Tanzania [6, 10]. A descriptive qualitative research design [12] was employed to explore mothers’ and fathers’ perceptions and experiences of humanizing birth care in Tanzania. The study was conducted in the two regional referral hospitals in the Mwanza and Mara regions in the Lake Zone of Tanzania that provide both basic as well as comprehensive obstetric and newborn care. Sekoutoure is the regional referral and teaching hospital in the Mwanza Region, which receive patients from Ukerewe, Misungwi, Kwimba, Magu, Sengerema, Ilemela and Nyamagana districts of Mwanza region [13]. It has 315 beds with 71 specifically for maternity services. Serving a population of 706,035 with an average of 30–40 deliveries daily [13]. The Musoma regional referral hospital in Mara region has 300 beds and serves a population of about 2 million [14]. The maternity ward has 20 delivery beds with an average of 14 deliveries daily [14].

Regional hospitals in Tanzania are the third level in the healthcare system referral pyramid where essential drugs, equipment, and skilled staff are supposed to be available to provide comprehensive emergency obstetric care and a wide range of maternity and child health services. The Lake Zone was chosen because it has the highest maternal mortality rates in the country. According to the 2012 census [15], Mara region has a maternal mortality ratio of 362 per 1000 births while the Mwanza region has a maternal mortality rate of 305 per 1000 births. These maternal mortality ratios are higher than those reported in other regions in Tanzania. For example, in 2015, Dar es Salaam (the commercial capital) had 137 maternal deaths per 1000 births with low maternal mortality ratios reported in Pwani (30 per 1000 births), Arusha (32 per 1000 births) and Lindi (39 per 1000 births) [16].

### Informants and data collection

Mothers and fathers who were not necessarily related and were available at the postnatal clinic during data collection period were recruited. The nurse midwife in charge of the clinic was requested to identify mothers who met the inclusion criteria and fathers were approached by the researchers. Those who met the inclusion criteria and agreed to take part in the study were recruited. Fathers were required to speak Kiswahili and had a wife who had recently given birth. Mothers were also required to speak Kiswahili and had to have two or more spontaneous normal vaginal deliveries to ensure that they have enough experience of giving birth in the health facilities. Most mothers and fathers were originally from the Lake Zone but from different sociodemographic areas were informed about the purpose and the method of the study including issues of confidentiality and the voluntary nature of their participation. Mothers and fathers were recruited until saturation of data was reached [17], when answers seemed to repeat information gained earlier and little new information was forthcoming.

To explore the perceptions and experiences of humanizing birth care in Tanzania, two methods of data collection were used: individual semi-structured interviews and focus group discussions (FGDs). Twelve (12) individual semi-structured interviews were conducted, 6 with fathers and 6 with mothers. While interviews with mothers and fathers at Sekoutoure hospital were conducted by the first and second author (LTM and TWK), those at Musoma hospital were conducted by nurses with Masters’ degree experienced in conducting health research. Interviews were conducted using a guide (Additional files 1 and 2) that was flexible and was revised in the course of data collection to allow new emerging issues to be included**.** The guide focused on participants’ experience of humanizing birth care when attending a healthcare facility for birth care. Although mothers and fathers could speak other languages including Kisukuma, Kikurya, Kiikizu, and Kijita, Kiswahili was used during data collection because it is the national language which was spoken fluently by all participants and data collectors. All interviews were held in a quiet, private, and convenient place that was identified by mothers and fathers, including their homes or around the hospital premises. All participants agreed to the use of an audio-recorder and interviews lasted between 30 and 40 min.

Four FGDs were conducted, two with mothers and two with fathers. The FGD with mothers had a total of 13 participants and that of fathers had 12 participants. FGDs was used to collect information on perceptions and experiences about humanizing birth care from mothers and fathers because of its ability to open up discussion without personalising opinions [18]. Before the discussion, ground rules were set whereby mothers and fathers were asked to respect each other’s opinion. They were informed that the discussion is confidential and they should not share information outside the group. They were also encouraged to actively participate during the discussion. Discussions were conducted in the hospitals setting and were moderated by the researchers (LTM and TWK) and research assistants using the Kiswahili language. The moderator was assisted by another researcher who took notes and made observations during the discussion. Each FGD took approximately 45 min and were audio-recorded with permission from mothers and fathers.

### Data analysis

Semi-structured interviews and FGDs were transcribed verbatim into Kiswahili and thereafter translated into English by hired research assistants fluent in both languages. Translation of interviews was necessary to ensure that non-Kiswahili speaking researchers could participate fully in the analysis process. Bohren’s et al. [11] typology on the mistreatment of women during childbirth in health facilities globally guided the analysis. The initial analysis sought to identify relevance of the participants’ account to a priori third order themes: (1) physical abuse, (2) sexual abuse, (3) verbal abuse, (4) stigma and discrimination, (5) failure to meet professional standards of care, (6) poor rapport between women and providers, and (7) health system conditions and constraints. For the analysis, all English transcripts were reviewed for first order themes and labelled accordingly, which were reviewed and re-labelled if necessary [19]. The second step involved reviewing each of the first order themes and quotes to categorize them based on the second order themes. The third step involved reviewing the second order themes and ensuring they fit into the third order themes. Using this approach, not all themes at each level that were outlined by Bohren et al. [11] emerged from our sample. During the second step, as long as one of the first order themes that corresponded to the second order themes were identified, it was considered present in our sample. For clarity, all identified themes at each of the orders are identified as present or absent in Table 2. To ensure agreement, the research team reflected, discussed differences, and agreed on mapping of codes onto the pre-existing framework. There was no quote identified that did not map onto an existing theme or code.

### Ethical consideration

This study was approved by the National Institute of Medical Research in Tanzania (Ref. no. NIMR/HQ/R.8a/Vol.IX/2143). All mothers and fathers signed the consent form to be interviewed after they were told about the purpose of the study. They were also assured about confidentiality of the information they provided and reminded that they should not share the information with other people. Each mother and father was given a unique identification number to provide anonymity during transcription and to ensure direct quotations were not attributable to individuals. All participants were informed that interviews and FGDs would be recorded and agreed for their anonymous quotes to be used.

## Results

Mothers’ age ranged from 20 to 45 years, whereas fathers’ age ranged from 25 to 60 years. Majority of mothers had at least primary education, were mainly housewives. Fathers on the other hand had at least a primary education level and worked as motorbike drivers. Most of mothers (*n* = 8) and fathers (*n* = 22) had three or more children (see Table 1).

**Table 1:** Characteristics of participants in the study

Factor	Category	Participants in the SSI		Participants in the FGDs (*n* = 25)	
		(*n* = 12)	%	*N* = 25	%
Age at interview	20–30	5	42	7	28
	31–40	3	25	11	44
	≥41	4	33	7	28
Education level	No education	1	8	1	4
	Primary	8	67	15	60
	Secondary or above	3	25	9	36
Occupation	Housewife	4	33	9	36
	Business	2	17	6	24
	Cashier/accountant	1	8	1	4
	Motorbike/bus driver	4	33	5	20
	Fisherman/peasant	1	8	2	8
	Teacher	0	0	2	8
Number of children	1–2	4	33	3	12
	3–4	7	58	19	76
	5–6	1	8	3	12

The findings from our study have been presented based on the Bohren’s et al. typology of mistreatment of women during childbirth [11] (see Table 2). This study found that mothers in Tanzania experienced verbal abuse, failure to meet professional standards of care, poor rapport between women and providers, and health system conditions and constraints. This study did not find experience of sexual or physical abuse nor stigma and discrimination to be prevalent.

**Table 2:** Typology of the mistreatment of women during childbirth according to Bohren et al. based on presence/absence in our findings

Third Order Themes		Second Order Themes		First Order Themes	
Present	Absent	Present	Absent	Present	Absent
	Physical abuse		Use of force Sexual abuse		Women beaten, slapped, kicked, or pinched during delivery Women physically restrained to the bed or gagged during delivery
	Sexual abuse		Sexual abuse		Sexual abuse or rape
Verbal abuse		Harsh language Threats and blaming		*Harsh or rude language* ^a^ Judgmental or accusatory comments	Threats of withholding treatment or poor outcomes Blaming for poor outcomes
	Stigma and discrimination		Discrimination based on socio-demographic characteristics Discrimination based on medical conditions		﻿Discrimination based on ethnicity/race/ religion Discrimination based on age Discrimination based on socioeconomic status Discrimination based on HIV status
Failure to meet professional standards of care		Lack of informed consent and confidentiality Physical examinations and procedures Neglect and abandonment		Refusal to provide pain relief Performance of unconsented surgical operations Neglect, abandonment, or long delays *Skilled attendant absent at time of delivery* ^a^	Lack of informed consent process Breaches of confidentiality Painful vaginal exams
Poor rapport between women and providers		Ineffective communication Lack of supportive care Loss of autonomy		Poor communication *Lack of supportive care from health workers* ^a^ Denial or lack of birth companions *Denial of food, fluids, or mobility* ^a^ Denial of safe traditional practices Lack of respect for women’s preferred birth positions	Dismissal of women’s concerns Poor staff attitudes Language and interpretation issues Women treated as passive participants during childbirth Objectification of women Detainment in facilities
Health system conditions and constraints		Lack of resources Facility culture	Lack of policies	Physical condition of facilities Supply constraints Bribery and extortion Unclear fee structures	Staffing constraints Staffing shortages *Lack of privacy* ^a^ Lack of redress Unreasonable requests of women by health workers

^a^indicates areas of treatment whereby mothers identified positive and/or respectful treatment in this domain

### Verbal abuse

Bohren et al. [11] identified verbal abuse as a third level theme of which mothers and fathers revealed experiencing either harsh or rude language as well as judgmental or accusatory comments that made them shamed or humiliated by healthcare providers. These map onto the second order themes of harsh language, threats, and blaming.

Some mothers and fathers reported experiencing harsh or rude language from healthcare providers. Both fathers and mothers recognized that healthcare providers in hospitals are well trained and skilled, yet they struggled with the way that women were verbally mistreated during the birthing process. Mothers also felt ignored by healthcare providers and, as a result, felt that healthcare providers saw them as a nuisance and bother.

“(...) hospitals are safe places with reliable tools for delivery of babies … nevertheless; there are minor challenges from health attendants with harsh and abusive words” (Father, FGD).

Mothers and fathers also experienced negative judgmental or accusatory comments during the birth process. Mothers felt blamed for becoming pregnant, not coming early enough to the hospital for delivery, or even for the birth process itself. Some mothers felt insulted by nurses and that they could not ask for help from the nurses during the delivery of their babies.

“She took the baby and put on the weighing scale and after that, she placed my baby on the bed and then told me to clean the place up because I was the one who messed up the place. I collected my clothes and then came back and cleaned up the place. By this time, the nurse was done with my baby so I just went to take shower and went back to sleep. I wasn't happy at all that I gave birth on my own”. (Mother, FGD)

“Honestly I was not pleased with the way we were welcomed. The health care provider kept on blaming [my wife] for not coming to the hospital early. All this time my wife was bleeding. I kindly asked for their help to stop the bleeding. Finally, they agreed and attended my wife”. (Father, FGD)

### Failure to meet professional standards of care

Bohren’s et al. [11] identified failure to meet professional standards of care as a third order theme. The first order themes identified within our study were refusal to provide pain relief, surgical operations performed without consent, neglect, abandonment, or long delays, and skilled attendant absent at time of delivery. These map onto the second order themes of lack of informed consent and confidentiality, physical examinations and procedures, and neglect and abandonment.

Mothers reported receiving unconsented surgical operations, being stitched without anaesthesia, and being refused pain relief when requested. One mother explained how she received both unconsented operations and was not given pain relief.

“( … ) if they had responded in time, maybe my parts wouldn't have been torn. Despite the fact that I was torn, they still stitched me without any pain killer and when I tried to refuse, I was told that I did not bring the required drugs and that if I did not want to be stitched without pain killer I should pay money for the drug and wait for them to go and buy the drugs. So to be honest, I will never ever return to that hospital again”. (Mother, FGD)

Mothers and fathers also reported that during the delivery process at a health facility, they experienced neglect, abandonment, and long waiting times. This was particularly experienced at the reception, where women struggled to be admitted on time due to delays at reception, which was beyond their control. Mothers and fathers reported that sometimes births occurred at the reception due to the delay in the process of admission into the labour ward that brings fear of giving birth at the reception.

“( … ) by the time I decide to go to the hospital with my relative, the process of admission at the reception is usually prolonged to the extent that I was afraid that something might happen to me. My opinion is that they should pay prompt attention to us and not keep us waiting because we are in pain and anything could happen to us”. (Mother, FGD)

Mothers reported being neglected during the delivery process while in the labour ward and others gave birth without a skilled healthcare provider being present. Mothers expressed that they felt that not all health care providers were interested in assisting their delivery process and were reluctant to help. Mothers often felt ignored during the delivery process, where they would ask healthcare providers for help but received no response.

“The nurses are used to the screams from the women in labour pain so they don’t pay much attention. However, not all women who are screaming are pretending. There are some of us who were in real pain. You may find women coming for the first time for delivery having a hard time because they don't know what to do. It’s really painful. So, if someone feels that the baby is coming and it’s time for delivery, she may call for help but only to be disappointed by nurses who think that she is pretending. But because they are busy with their own things, they don't pay attention. When my time reached, I went to call the nurse but she didn't pay any attention. They were calling me ‘that troublesome woman’ and wanted me to stop bothering them. But there was a doctor who was passing by and asked why they were chatting while I was calling for help. When they came, they saw that the baby’s head had started coming out and immediately started assisting me. I wonder if that doctor had not passed by, I would have delivered alone. I was really mad at this point”. (Mother, SSI)

The experience of neglect by healthcare providers was also shared during group discussion:

“I gave birth at the hospital but I would not want to return to that place again because I had a terrible experience. When I called them for help, they ignored me. Until the last minute, I told them that the baby was nearly coming but they still ignored me until the baby came forcefully and my parts were torn”. (Mother, FGD)

### Poor rapport between women and providers

Another third order theme that was identified by Bohren et al. [11] and present in our findings was poor rapport between mothers and providers. The first order themes identified were poor communication, lack of supportive care from health workers, denial or lack of birth companions, denial of mobility and safe traditional practices, and lack of respect for women’s preferred birth positions. These map onto the second order themes of ineffective communication, lack of supportive care, and loss of autonomy.

Mothers and fathers experienced poor communication. For instance, mothers were not informed when healthcare providers took their babies to measure weight after delivery. Fathers, on the other hand, were left out of the communication regarding their wives’ birth process. They were not allowed into the delivery room and either had to wait outside or at home. A father described how he struggled to find information about his wife’s progress while she was at the labour ward:

“Sometimes you face problems. If you want to know about what is going on with your wife … if you do not see your wife during visiting time, you have to go back home and wait for another visitation time. You are not given any information neither can you call your wife because at the time, she is told not to use her phone to communicate unless there is an emergency”. (Father, SSI)

Denial or absence of companions at birth was also reported. Fathers are rarely allowed into the delivery room, yet mothers expressed a desire for husbands to be more involved in the delivery process. Traditionally, husbands have not been involved with maternal health and the birth experience. Mothers and fathers expressed that the hospital culture and physical infrastructure limits the ability for fathers or other companions including mother-in-laws to be involved. The increased desire for husbands to actively be involved during the delivery process was associated with promotion of equality between the husband and wife in the family such that the husband is aware of what the wife experienced during delivery and was involved in making informed decisions on family planning.

“I would love my husband to be present at the time of delivery. I would love for him to witness the kind of turmoil we ladies go through during delivery so that he can be more cooperative. Because you may find that some men don't offer full support to their wives during pregnancy and even delivery because they don't understand how difficult it is. But if he is present and sees how tough it gets, maybe he will be more supportive. This might change his attitude a little bit and understand that his wife endures a rough time during pregnancy and labour. This might instil a sense of solidarity and supportiveness towards me. I would really like him to be present during delivery time”. (Mother, SSI)

Mothers reported experiencing loss of autonomy when healthcare providers prevented them from moving around and did not allow them to take food or fluids during labour. They were also denied the use of safe traditional practices while in the hospital, yet many also asserted that this was expected and not necessarily an issue to them. They recognize that hospitals are dominated by science and technology hence traditional practices are either conducted at home before arriving at the hospital or would be completed at home after the birth, if at all.

“When I am at home, I do them and before I take her to the hospital, I perform some rituals and when she returns home I will, but I won’t bother the hospital staff with my personal traditions”. (Father, SSI)

Mothers also reported lack of respect for preferred birth positions with doctors and nurses encouraging the use of supine position over anything else. Mothers even reported not knowing that any other position was possible due to previous healthcare facility births where supine position was the only option. Mothers, however, reported preferring other positions, including the squatting position, which was discouraged during healthcare facility birth.

“I would prefer to select the birth position but she [the nurse] said that because it’s my first pregnancy, I should get used to delivering while on my back”. (Mother, SSI)

“( …) I was lying on my back and I had pulled my legs raising my knees. If I was allowed to choose, I would have delivered while sitting”. (Mother, SSI)

### Health system conditions and constraints

The last third order theme identified by Bohren et al. [11] that was present in our findings was health system conditions and constraints. The first order themes identified in this study were physical condition of health facilities, supply constraints, bribery and extortion, and unclear fee structures. This maps onto the second order themes of lack of resources and facility culture.

Mothers and fathers were concerned about the physical condition of the hospital. They reported that mothers often shared beds, were discharged early due to lack of space, or mothers delivered on the floor due to lack of available beds. They were asked to buy supplies, such as gloves and syringes, and were turned away if they did not have these requirements. If an episiotomy was required, they would have to bring anaesthesia prior to delivery or else risk being stitched without anaesthesia, as explained earlier.

“( … ) a pregnant woman might arrive at the hospital to delivery but unfortunately, a bed might at that time be occupied due to large number of women admitted in the labour ward waiting for delivery or more than one woman might share one bed”. (Father, SSI)

Both fathers and mothers had concerns about the facility culture, particularly bribery, extortion and unclear fee structure. Maternity care in Tanzania is supposed to be free yet several mothers and fathers reported being asked for additional payments or to pay bribe:

“We learnt through the media and government outlets that maternity services are free of charge, but they wanted to make me pay for the bed. I didn't pay but it was after a lengthy argument that they decided to just let my wife get a free bed”. (Father, SSI)

### Positive treatment

While there was substantial description of mistreatment experienced by mothers in this study, some mothers experienced positive treatment by healthcare providers. Mothers received encouraging words, healthcare providers who provided instructions about what to do during labour, and encouraged deep breathing. Some mothers also commented that they were offered support during all stages of delivery and mentioned the benefits of having a skilled healthcare provider present at birth:

“The information is meaningful. For example, when they tell you that now you are ready [to deliver], you set your mind and body for action so that I get done with this and then I can rest later. Or when there is still time, they can tell you to go back and sleep. Even after giving birth, they congratulate you and tell you the kind of baby, if it’s a boy or girl. I was really happy with the information that I was being given”. (Mother, FGD)

“I felt great because after just giving birth, the nurse consoled me for what I had gone through and the pain that I had endured earlier. So, I felt at peace. It feels really good when the nurse congratulates, and consoles you for what you have been through, it feels really great. I had no problem, I felt very well”. (Mother, FGD)

During interviews, the experience of receiving positive interactions from doctors and nurses was shared:

“The doctor is the one who assisted me. When the doctor examined me, he found out that the baby was distressed and he told me that a normal delivery would be very risky to both the baby and me. So, they took me to the theatre and operated on me to save both our lives … I got a lot of assistance, for instance, there was a time when I couldn't breathe and they placed an oxygen mask on me. Nurses were constantly speaking such that I didn't fall asleep [during surgery]. They were very kind to me, they were telling me some jokes, and they were very cooperative to me. I really enjoyed the experience”. (Mother, SSI)

Mothers also felt that their privacy was respected, both in terms of physical privacy during the birth process as well as confidentiality among the staff:

“It was just the nurse and I [in the delivery room]. I would like to be alone with the nurse. I think that is proper”. (Mother, SSI)

## Discussion

Mothers in Tanzania do not always receive respectful maternity care when delivering in a health facility, indicating that they may experience verbal abuse, a failure to meet professional standards of care, poor rapport between women and providers, and limiting health system conditions and constraints. However, not all birth experiences have been negative, with mothers identifying areas where they received respectful maternal care. Using Bohren et al. [10] themes of disrespectful care, this study was able to map the experiences of respectful maternity care in two regions of the Lake Zone in Tanzania as experienced by mothers and fathers.

It is clear that there are areas where mothers continue to face mistreatment and disrespectful maternity care during childbirth in Tanzania. In this study, we identified that mothers were talked to in harsh or rude language and that judgmental or accusatory comments made them feel shamed or humiliated by healthcare providers. Mothers also were refused pain relief and had unconsented surgical operations. They also experienced neglect, abandonment, or long delays and skilled attendant were sometimes absent at time of delivery. They also experienced poor rapport between themselves and healthcare providers, including poor communication and a lack of supportive care. They were also denied the presence of their husbands as birth companions as well as denied mobility and safe traditional practices. They were also denied the choice of their preferred birth positions. Finally, they were impacted by the poor physical condition of facilities and supply constraints, and as a result, experienced bribery, extortion, and unclear fee structures.

These themes of disrespect and abuse during childbirth have continued to be echoed in other studies around the world [20–22]. In Uttar Pradesh, India, all women experienced at least one aspect of mistreatment, predominant denied their preferred birth position [23]. Looking specifically at Tanzania, a study conducted in the Morogoro region similarly found that men identified harsh and disrespectful care received by their wives during or after childbirth, including feeling ignored, neglected or mistreated, experiencing concerns about money, and experiencing verbal abuse [24]. Furthermore, women in the Tanga region who experienced disrespect and abuse during childbirth described low satisfaction and low quality of care, which resulted in them being unlikely to deliver at the same facility with their next child [25]. Even in the urban areas of Dar es Salaam where it is likely that there are more skilled healthcare providers and resources, women continue to experience disrespect and abuse during childbirth. A recent study by Sando et al. [26] found that 15% of women reported experiencing some form of disrespectful or abusive behaviour when interviewed three to 6 hours postpartum, which increased to 70% when they were followed up weeks later. It is overwhelmingly clear that challenges remain in humanizing the birth process in Tanzania.

As reported by the participants in this study, there is a desire by both fathers and mothers to have the father more involved when the mother is giving birth, yet due to space limitations within the hospital ward, privacy, mistrust of men by nurses, and systematic traditions whereby fathers were not previously involved, their ability to be involved continues to be limited. Fathers continue to be told to wait in the reception area while their wives go through labour, despite their desire to be involved in the delivery process [6]. Fathers also expressed that they felt disconnected from the delivery process due to a lack of communication from the healthcare providers at the hospital. In order for fathers to increasingly be involved in maternal health care, traditional gender norms and institutional barriers need to be removed [27]. However, the concept of the labour room as a space for mothers only continues to dominate the discourse in several low resource countries [28]. Previous work carried out in Tanzania identified barriers to male involvement in pregnancy and delivery to include: traditional gender roles, low levels of knowledge, perceived lack of ability to partake in antenatal care visits, and prior negative experiences in health facilities [27]. Furthermore, the facility’s physical infrastructure limits the ability for fathers to be present at the bedside during labour as many hospital have limited physical space and are too overcrowded already without having additional people in the labour room [29]. Therefore, future work is required to identify ways to better accommodate fathers during maternity care to encourage greater respectful care.

What is interesting is that there were mixed findings when comparing the perspective of fathers and mothers. For instance, both mothers and fathers reported negative experiences related to verbal abuse during the delivery period, but interestingly only mothers reported positive experiences by healthcare providers where they received encouragement rather than abuse. This could in part be related to the inability for fathers to be fully involved throughout the delivery process, whereby fathers are excluded from the labour ward and forced to wait either at home or in the waiting room [6]. In humanizing the birth process, it is essential that these differences are taken into account to ensure that the rights and desires of both the mother and father are taken into account and respected.

It is interesting to explore the themes that were not mentioned in our study including; sexual abuse, physical abuse, and stigma and discrimination. Looking specifically at physical abuse, a recent study explored disrespect from the perception of nurse midwives in urban Tanzania through naturalistic observation identified that physical abuse occurred, including slapping and hitting but also incorrect provision of drugs or care [30]. This disconnect is perhaps related to the methodology used, whereby some mothers and fathers may not feel comfortable disclosing to an interviewer or during a focus group that they experienced physical or sexual abuse. However, other studies in Tanzania have acknowledged that physical abuse is rarely mentioned in Tanzania [24]. Further work is needed to continue to explore the gaps in care that mothers receive during delivery in Tanzania particularly as it relates to these vulnerable areas of care.

Despite the gaps and negative experiences described, some of the areas where mothers reported positive birthing experiences and respectful maternity care included: continuous support during childbirth (including having a skilled attendant help with her birth), having freedom of movement during labour (particularly around exercise), good communication between client and provider (including encouragement during the birthing process), support of the mother-baby-pair before and after delivery (including privacy received during delivery). It is encouraging that some mothers reported positive experiences or moments of care during a health facility delivery as every woman has the basic human right to respectful maternity care [20]. In a review on women’s satisfaction with maternity health care across all developing countries, Srivastava et al. [31], identified that the quality of care was the most important factor for maternal satisfaction, including receiving supportive and non-abusive interpersonal behaviour from nurses. Structural factors related to good physical environment, cleanliness, and availability of adequate healthcare providers, medicines, and supplies as well as process factors such as privacy, promptness of care, and emotional support were desired [31]. There is a need for learning from countries that offer respectful maternity care [21, 32] to guide future policies and research to ensure that all Tanzanian mothers received respectful maternity care.

To further humanize the birth process in Tanzania, it is essential that greater focus is put on changing the culture around respectful maternal care. This must include shifting the culture of healthcare providers to be more mother-focused, rather than institution-focused, whereby mothers’ desire for increased father involvement and continuous high-quality care is respected and provided [33, 34]. Health systems and provider behaviours need to create an environment where all women have access to respectful, competent, and caring maternity health care services [20]. In the seminal article by Wagner [1] on the need to humanize birth, key strategies identified to humanize birth include: the education of both healthcare provider and women to be able to make informed decisions; the promotion of evidence-based maternity practices; empowering midwives to provide primary maternal care; and to integrate out-of-hospital and in-hospital birth care and practitioners. Based on our findings and others [33], there is much work that needs to be done to humanize the birth process in Tanzania. Tanzania has a strong midwifery history which can and should be utilized. By expanding the midwifery and nursing education to include information on respectful maternal care and the use of evidence-based maternity practices, it may increase the likelihood of women receiving respectful maternal care. If midwifery education was to emphasize humanizing birth in its curriculum, it could lead the way in humanizing birth care in developing countries.

### Strength and limitations

This qualitative study was conducted with rigour. Trustworthiness of the data was established using various methods including validation of the key themes through dialogue with all members of the research team, as well as continuous reflection and revisiting codes to ensure accurate fit [17]. To ensure authenticity and criticality, this study used multiple data sources (i.e., mothers, fathers) and methods (i.e., semi-structured interview, FGD) to allow for triangulation of results, which is important in qualitative research to increase credibility [17].

Nevertheless, there are some limitations to this current study. One limitation is that the analysis of the FGDs and semi-structured interviews were completed in English from translated transcripts. However, the transcripts were verified by co-authors fluent in Kiswahili to ensure adequate translations and all codes and themes were discussed amongst the co-authors who were able to review the original transcripts. Additionally, the fact that mothers and fathers were interviewed at a postnatal ward where they were continuing to receive care may have limited their willingness to be completely open and honest with the interviewers on their birthing experience. As evident in the study by Sando et al. [26], women were significantly more likely to report disrespect and abuse when interviewed in their homes weeks after their childbirth experience compared to hours post-partum. To mitigate this, the interviews were conducted by healthcare providers outside the health facility and interviews were held in a private area. Furthermore, because mothers and fathers also shared their negative experiences, we believe the location was less likely to be a hindrance to our findings. These results can be relevant to other countries with similar social-economic characteristics as that of Tanzania.

## Conclusion

Despite the increasing number of deliveries occurring in health facilities, there continue to be challenges in providing respectful maternal care with mothers continuing to receive mistreatment during childbirth. Humanizing birth care in Tanzania has a long way to go but there is evidence that changes are occurring as women are noticing and reporting positive changes in delivery care practices. There is need and opportunity to train the next generation of healthcare providers on respectful maternity care and the use of evidence-based maternity practices, which should lead towards a significant shift in humanizing birth care in Tanzania.

## Additional files

Additional file 1: Semi-structured interview guide for fathers. (DOCX 23 kb)

Additional file 2: Semi-structured interview guide for postnatal mothers. (DOCX 22 kb)

## Data Availability

The datasets used and/or analysed during the current study are available from the corresponding author on reasonable request.
