# Supplementary material for: Humanizing birth in Tanzania: a qualitative study on the (mis) treatment of women during childbirth from the perspective of mothers and fathers
Source: BMC Pregnancy Childbirth. 2019 Jul 5;19:231. doi: 10.1186/s12884-019-2385-5 (PMC6612108; doi:10.1186/s12884-019-2385-5)
Supplement: Supplementary file 1 — Semi-structured interview guide for fathers. (DOCX 23 kb) [file 12884_2019_2385_MOESM1_ESM.docx]

**Semi Structured Interview Guide for Fathers**

| Date of Interview |  |
| --- | --- |
| Time of Interview |  |
| Code number |  |
| Region |  |
| District |  |
| Village and ward |  |

**Part A: Social demographic information**

| 1. Age |  |
| --- | --- |
| 1. Education level |  |
| 1. Occupation |  |
| 1. Parity |  |

**Part B: Questions addressing objectives**

1. Could you please tell me where did your wife/partner give birth?

Probe

- Who chose that place for her?
- What facilitated the choice to the place of delivery?
- If you had a chance to choose would you choose the same place? Why?

1. What kind of support did your wife/partner receive when she was in labour?

Probe:

- Who supported her?
- What was your expectation?

1. Which position did your wife/partner assume during delivery? Did she told you why opted such position?

Probe:

- Who decided that position for her?
- If you had chance to discuss with your wife/partner on choice of delivery positions, which one would you suggest? Why?
- During labour what was your wife/partner’s experience regarding free movement during labour?

1. How would you have been like to be involved during your wife/partner’s delivery experience?

Probe:

- Were you present?
- Who did you like to be present during labour and delivery? Why?

1. How were your beliefs considered during labour and delivery?

Probe:

- Cultural, spiritual and tradition

1. How well were you informed of the laboring and delivery process of your wife/partner in each step?
2. How well were you satisfied with the care given to your wife/partner by the care providers?

Probe:

- Right time?
- Correct information?
- Usefulness?

1. What happened to your baby immediately after delivery?

Probe:

- Were your child given to your wife/partner immediately and stayed with the child throughout?
- What did you like to be done to your child after delivery?

1. In your opinion, was there anything that was done by care providers that was not appropriate for you?
