# Supplementary material for: Humanizing birth in Tanzania: a qualitative study on the (mis) treatment of women during childbirth from the perspective of mothers and fathers
Source: BMC Pregnancy Childbirth. 2019 Jul 5;19:231. doi: 10.1186/s12884-019-2385-5 (PMC6612108; doi:10.1186/s12884-019-2385-5)
Supplement: Supplementary file 2 — Semi-structured interview guide for postnatal mothers. (DOCX 22 kb) [file 12884_2019_2385_MOESM2_ESM.docx]

**Semi Structured Interview guide for Postnatal Mothers**

| Date of Interview |  |
| --- | --- |
| Time of Interview |  |
| Code number |  |
| Region |  |
| District |  |
| Health facility |  |

**Part A: Social demographic information**

| 1. Age |  |
| --- | --- |
| 1. Education level |  |
| 1. Occupation |  |
| 1. Parity |  |

**Part B: Questions addressing objectives**

1. Could you please tell me where did you give birth?

Probe

- Who chose that place for you?
- What facilitated the choice to the place of delivery?
- If you had a chance to choose would you choose the same place? Why?

1. What kind of support did you receive when you were in labour?

Probe:

- Who supported you?
- What was your expectation?

1. Which position did you assume during delivery?

Probe:

- Who decided that position for you?
- If you had chance to choose position which one would you choose? Why?
- During labour were you free to move around as you wished?

1. Could you please describe your delivery experience?

Probe:

- Who was present?
- Who did you like to be present during labour and delivery? Why?
- What support did you receive during delivery

1. How was the privacy maintained during labour& delivery?

Probe:

- How did you feel about your security and safety?

1. How did you feel during labour and delivery? Why?

Probe:

- Were you given chance to choose the provider that would assist you during labour and deliver?

1. How did your believes considered during labour and delivery?

Probe:

- Cultural, spiritual and tradition

1. How well were you informed of the laboring and delivery process in each step?
2. How well were you satisfied with the information you received from the care providers?

Probe:

- Right time?
- Correct information?
- Usefulness?

1. What happened to your baby immediately after delivery?

Probe:

- Were you given your child immediately and stayed with it throughout?
- What did you like to be done to your child after delivery?

1. In your opinion, was there anything that was done by care providers that was not appropriate for you?
